# Early Echocardiographic Changes Following Transcatheter Aortic Valve Implantation: A Comparative Analysis of Different Transcatheter Aortic Valve Systems

**DOI:** 10.3390/jcdd13050173

**Published:** 2026-04-22

**Authors:** Huseyin Dursun, Tugce Colluoglu, Bihter Senturk, Hatice Ozdamar, Cisem Oktay, Hacer Uysal, Husna Tugce Simsek, Zulkif Tanriverdi, Dayimi Kaya

**Affiliations:** 1 Department of Cardiology, Faculty of Medicine, Dokuz Eylül University, Izmir 35340, Turkey; drhuseyindursun@gmail.com (H.D.); drbihter@hotmail.com (B.S.); cisem.oktay@deu.edu.tr (C.O.); hacer.uysal@deu.edu.tr (H.U.); husnatugce.simsek@deu.edu.tr (H.T.S.); dayimikaya@gmail.com (D.K.); 2 Department of Cardiology, Tepecik Training and Research Hospital, Izmir 35020, Turkey; 3 Department of Cardiology, Faculty of Medicine, Harran University, Sanliurfa 63300, Turkey; ztverdi@gmail.com

**Keywords:** transcatheter aortic valve implantation, TAVI valves, two-dimensional echocardiography, valve hemodynamics

## Abstract

Background: Transcatheter aortic valve implantation (TAVI) is a viable alternative therapeutic approach for patients with severe aortic stenosis (AS), following technological innovations in transcatheter aortic valve systems and advances in clinical expertise, which aim to optimize valve hemodynamics. In this study, we aimed to compare early hemodynamic changes in different types of TAVI valves via two-dimensional echocardiography. Methods: This retrospective observational study examined patients with severe AS who underwent transfemoral TAVI. Patients were classified according to expansion mechanism (self-expanding valves (SEVs) or balloon-expandable valves (BEVs)) and leaflet position relative to the annulus (supra-annular valves (SAVs) or intra-annular valves (IAVs)). The implanted prostheses were Edwards SAPIEN XT valves (ESV, Edwards Lifesciences, Irvine, CA, USA), Medtronic valves (Core Valve-MCV and Evolut R, Medtronic, Minneapolis, MN, USA), Portico valves (St. Jude Medical, Saint Paul, MN, USA), and Myval valves (Meril Life Sciences, Vapi, India). Baseline two-dimensional transthoracic echocardiography (TTE) datasets were compared with post-TAVI measures obtained before discharge. Results: In total (*n* = 332), 275 (82.8%) patients were treated with SEVs, and 57 (17.2%) were treated with BEVs. In terms of leaflet position, 249 (75%) patients were treated with SAVs, and the remaining 83 (25%) patients were treated with IAVs. Transaortic gradients were comparable between patients treated with SEVs and BEVs. However, patients treated with IAVs exhibited significantly higher aortic maximum gradients (16 [13–21] mmHg vs. 14 [10–20] mmHg, *p* = 0.019) and mean gradients (9 [7–11] mmHg vs. 8 [5–10] mmHg, *p* = 0.014) compared to those receiving SAVs. Post-TAVI gradients were also compared based on each TAVI device. Although post-TAVI aortic maximum gradient was comparable among TAVI devices (*p* = 0.080), aortic mean gradient was significantly different among the valves (*p* = 0.006). Post hoc analyses demonstrated that the post-TAVI mean gradient was significantly lower in Medtronic CoreValve compared to the Myval (*p* = 0.013) and Portico (*p* = 0.030). No significant differences were observed in the frequency of perivalvular leak between the valve groups. Conclusions: We found that post-TAVI transaortic gradients of SEVs and BEVs were comparable; however, SAVs were associated with lower transaortic gradients than those of the IAVs. In addition, the frequency of ≥moderate PVL was comparable between the valve groups.

## 1. Introduction

Calcific aortic valve stenosis (AS) is the most prevalent valvular heart disease in individuals over 75 years of age, with aortic valve calcification detected in approximately 26% of this population [1]. Transcatheter aortic valve implantation (TAVI) has emerged as the preferred therapeutic strategy for elderly patients with symptomatic, severe AS who are at high risk for surgery. Subsequently, it has been established as an evidence-based alternative to surgical aortic valve replacement in patients with intermediate and low surgical risk [2,3,4].

The first-generation self-expanding, supra-annular Medtronic Core Valve (MCV, Medtronic Inc, Minneapolis, MN, USA) and the intra-annular balloon-expandable Edwards SAPIEN XT valves (ESV, Edwards Lifesciences, Irvine, CA, USA) are the predominant TAVI valves in use [5]. Early-generation devices have inherent limitations, notably perivalvular leak (PVL), which considerably reduces the efficacy and safety of TAVI [6]. To overcome the shortcomings of older-generation devices and yield improved results, many new-generation transcatheter aortic valve systems have been implemented extensively in clinical practice [7]. The global utilization of TAVI has expanded substantially, with the number of annual procedures projected to increase from nearly 100,000 in 2017 to an estimated 300,000 by 2025 [4]. However, contemporary TAVI valve platforms differ in expansion mechanisms, stent frame architectures, and leaflet design—features that may influence both short-term hemodynamic performance and long-term valve durability [4,8].

This study sought to compare four distinct TAVI valves, grouped in terms of their expanding mechanism (self-expanding valves [SEVs] vs. balloon-expandable valves [BEVs]) and leaflet position relative to the annulus (supra-annular valves [SAVs] vs. intra-annular valves [IAVs]), regarding their impact on early hemodynamic characteristics observed via transthoracic echocardiography (TTE).

## 2. Materials and Methods

### 2.1. Study Design and Data

This retrospective, single-center observational study focused specifically on patients with severe AS who underwent transfemoral TAVI between 9 June 2012 and 1 January 2024. This study was approved by the local institutional ethics committee, and all procedures adhered to the ethical principles outlined in the Declaration of Helsinki.

All patients were evaluated by a multidisciplinary heart team including two cardiologists, two cardiac surgeons, and one cardiac anesthesiologist. The decision to perform TAVI was made in accordance with the European Society of Cardiology (ESC) guidelines on the management of valvular heart disease applicable at the time of each patient’s evaluation, encompassing the 2012, 2017, and 2021 ESC/EACTS guideline versions, given the study inclusion period spanning from 2012 to 2024 [2,9,10]. The diagnosis of severe AS was confirmed through a review of the patients’ echocardiographic reports, obtained from the local hospital’s electronic information management system. Severe AS was defined as an aortic valve area of <1.0 cm^2^ and/or a mean transaortic gradient of >40 mmHg. A low flow across the aortic valve was described as an indexed stroke volume < 35 mL/m^2^, and low-flow, low-gradient (LFLG) AS was defined as a mean transvalvular gradient < 40 mmHg, effective orifice area (EOA) < 1.0 cm^2^, and left ventricular ejection fraction (LVEF) < 40% [11]. Variables including age, sex, comorbidities, laboratory variables, and estimated Society of Thoracic Surgeons (STS) score were extracted from the hospital’s electronic information management system.

The TAVI valves implanted were ESVs, Medtronic valves (MCVs, Evolut R), Portico valves (St. Jude Medical, St. Paul, MN, USA), and Myval valves (Meril Life Sciences, Vapi, India). The type and size of each valve used in this study are shown in Table 1. Patients were divided into two specific groups, according to the expansion mechanism (SEVs (Medtronic and Portico valves) vs. BEVs (ESVs and Myval valves)) and leaflet annular position (SAVs (Medtronic valves) vs. IAVs (ESV, Portico valves, Myval valves)) [12,13].

### 2.2. Echocardiography

Standard TTE was performed before TAVI and before discharge from the hospital. The echocardiography database was used to identify the following measurements: LVEF, left ventricular end-diastolic diameter (LVEDD), left ventricular end-systolic diameter (LVESD), interventricular septum (IVS) thickness, posterior wall (PW) thickness, left atrium diameter (LA), pulmonary artery systolic pressure (PASP), maximum aortic jet velocity (AVmax), maximum aortic valve gradient, mean aortic valve gradient, aortic valve area (AVA), mitral regurgitation (MR), aortic regurgitation (AR), PVL, and tricuspid regurgitation (TR). Severity of PVL was classified using a four-grade scheme as follows: none/trivial, mild, moderate, and severe.

### 2.3. Statistical Analysis

All statistical analyses were performed using SPSS version 30.0 (IBM Corp., Armonk, NY, USA). The Kolmogorov–Smirnov test was used to assess the normality of the data. Continuous variables with a normal distribution were presented as means ± standard deviations and compared with the independent-sample *t* test for two independent groups. Continuous variables without a normal distribution were presented as medians (Q1–Q3) and compared with the Mann–Whitney U test for two independent groups. The Kruskal–Wallis H test was used for the comparison of more than two groups without a normal distribution. Pairwise Mann–Whitney test with Bonferroni correction was performed for subgroup analyses. Categorical variables were expressed as absolute numbers and percentages (%) and compared with the chi-square or Fisher’s exact chi-square test. Comparisons of pre-TAVI and post-TAVI characteristics were performed with a paired-sample *t* test for normally distributed variables and the Wilcoxon test for non-normally distributed variables. All *p*-values were two-sided and considered statistically significant when less than 0.05.

## 3. Results

A total of 332 patients (58% female) with severe symptomatic AS who underwent TAVI between 9 June 2012 and 1 January 2024 were retrospectively included in the analysis. Regarding the expansion mechanism, 275 (82.8%) patients were treated with SEVs, and 57 (17.2%) were treated with BEVs. In terms of leaflet position relative to the annulus, 249 (75%) patients were treated with SAVs, and 83 (25%) patients were treated with IAVs (Table 1). Peripheral artery disease was significantly more prevalent in patients with SAVs compared to those with IAVs (23 [9.3%] vs. 2 [2.4%], *p* = 0.040). Also, STS score tended to be higher in patients treated with SEVs than in BEVs (4.2 [3.0–5.9] % vs. 3.8 [2.6–4.9] %, *p* = 0.056). No significant differences were observed in the prevalence of other comorbidities or laboratory variables between patients implanted with SEVs and those implanted with BEVs or between those implanted with SAVs and those implanted with IAVs (Table 2).

Baseline echocardiographic linear dimensions, aortic valve gradients, AVA, PASP, and the degree of MR and TR were comparable across groups (Table 3).

Echocardiographic parameters obtained before discharge are listed in Table 3. The aortic maximum gradient tended to be lower in patients treated with SEVs than in BEVs (14 [10–20] vs. 16 [13–21], *p* = 0.055). Other echocardiographic variables were comparable between patients treated with SEVs and BEVs. However, patients implanted with IAVs exhibited significantly higher aortic maximum gradients (16 [13–21] mmHg vs. 14 [10–20] mmHg, *p* = 0.019) and aortic mean gradients (9 [7–11] mmHg vs. 8 [5–10] mmHg, *p* = 0.014) compared to those implanted with SAVs. Other post-TAVI echocardiographic measurements did not differ significantly between patients with IAVs and SAVs (Table 4).

**Figure 1:**
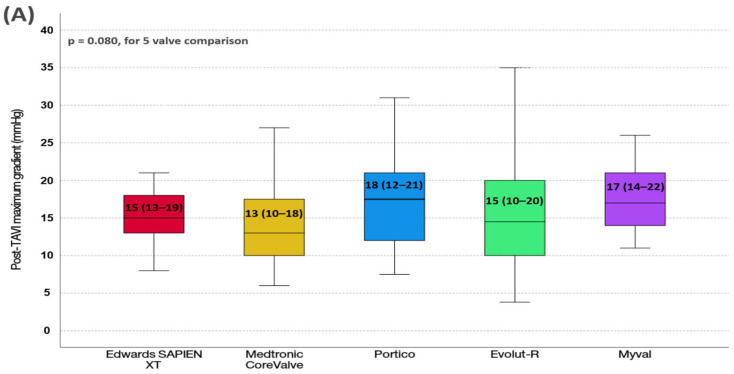

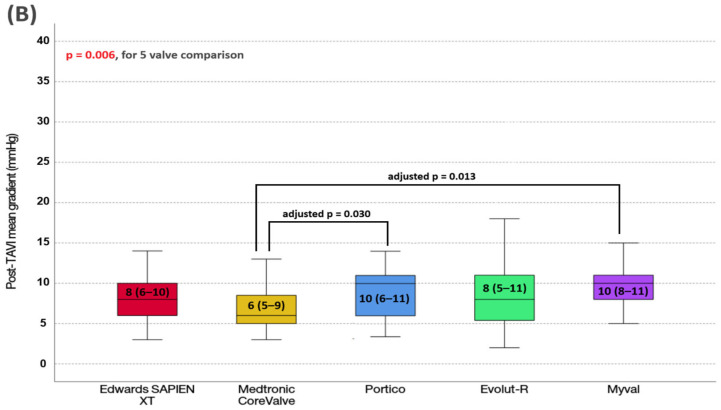
Comparisons of Post-TAVI (**A**) maximum and (**B**) mean transaortic gradients among TAVI devices.

Additionally, we compared post-TAVI gradients based on each TAVI device (Figure 1). We found that post-procedural aortic maximum gradient was comparable among TAVI devices (*p* = 0.080). However, post-procedural aortic mean gradient was significantly different among the valves (*p* = 0.006). The Medtronic CoreValve had the lowest post-TAVI mean gradient. Post hoc analyses demonstrated that the post-TAVI mean gradient was significantly lower in Medtronic CoreValve compared to the Myval (adjusted *p* = 0.013) and Portico (adjusted *p* = 0.030).

**Figure 2:**
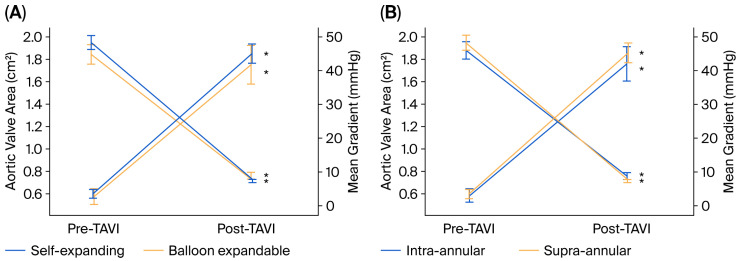
Pre-TAVI and post-TAVI comparisons of aortic valve area and mean transaortic gradients (**A**) between self-expanding and balloon-expandable valves and (**B**) between intra-annular and supra-annular valves. * *p* < 0.001 for pre- vs. post-TAVI.

The changes observed after TAVI for each group in terms of the mean gradient and AVA are shown in Figure 2. After TAVI, the mean gradient decreased, and AVA increased significantly in all groups (*p* < 0.001) (Figure 1). In addition, we found that LVEF was significantly increased after TAVI compared to pre-TAVI measurements (from 52.2 ± 13.6 to 55.1 ± 11.6, *p* < 0.001).

**Figure 3:**
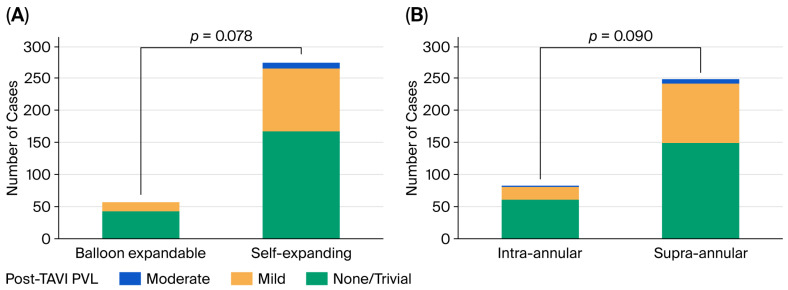
Comparison of the degree of perivalvular leak (**A**) between balloon-expandable and self-expanding valves and (**B**) between intra-annular and supra-annular valves. PVL, perivalvular leak.

No statistically significant differences were observed in the frequency of PVL between SEV and BEV patients or between SAV and IAV patients. Also, there was no significant difference among TAVI devices regarding PVL based on each TAVI device classification (*p* = 0.182). No patients had a severe PVL. Moderate PVL was observed in 3.3% of patients with SEVs, while no PVL was observed in patients with BEVs (Table 5 and Figure 3).

## 4. Discussion

This study is unique as it offers an early hemodynamic comparison of TAVI valves according to their expansion system (SEV vs. BEV) and leaflet position relative to the annulus (SAV vs. IAV). The main findings can be summarized as follows. First, we observed that aortic valve hemodynamics were significantly improved in each TAVI valve group shortly after TAVI. Second, aortic maximum gradients and aortic mean gradients were higher in patients with IAVs compared to those with SAVs. Third, in device-based comparison, Medtronic CoreValve had a significantly lower aortic mean gradient compared to the Myval and Portico. Finally, there were no significant differences in the frequency of PVL between patients with SEVs and BEVs or between SAV and IAV patients.

A thorough hemodynamic assessment following TAVI is strongly advised to predict the long-term performance of the implanted valve and clinical outcomes [14]. Growing evidence suggests that higher mean transvalvular pressure gradients after TAVI are a crucial determinant of long-term valve performance and clinical outcomes, as higher residual gradients have been associated with impaired left ventricular mass regression, accelerated structural valve degeneration, worse functional capacity, increased hospitalization, and reduced survival. Importantly, these hemodynamic differences appear to be influenced by the spatial position of the valve leaflets relative to the native annulus, highlighting the importance of supra-annular versus intra-annular valve design [15,16]. It has been demonstrated that post-TAVI transvalvular pressure gradients are lower when self-expanding supra-annular valves are used compared to balloon-expandable intra-annular valves, a difference that is particularly evident in patients with small aortic annuli [17,18]. This advantage is predominantly attributed to the supra-annular location of the functional orifice, which allows for a larger effective orifice area and reduces annular restriction [19]. In our study, although it did not reach statistical significance, the aortic maximum gradient tended to be lower in patients treated with SEVs than in BEVs. This may be due to the relatively small number of patients in the BEV group. If we had a slightly larger number of patients in the BEV group, this difference would be even more significant. In contrast, the residual gradients were significantly higher in IAV patients compared to SAV patients. Moreover, we compared post-TAVI gradients based on each TAVI device and found that the post-TAVI mean gradient was significantly lower in Medtronic CoreValve compared to the Myval and Portico.

The OPERA-TAVI registry, which evaluates the latest devices, recorded median mean gradients of 7.0 mmHg for SEVs compared to 12.0 mmHg for BEVs (*p* < 0.01) [20]. This difference in mean gradients persists across multiple studies and represents a clinically meaningful difference [21,22]. The randomized CHOICE trial reported mean gradients of 6.4 mmHg versus 8.4 mmHg at initial follow-up, with this hemodynamic superiority maintained at 5 years (6.9 mmHg versus 12.2 mmHg; *p* = 0.001) [23,24]. Meta-analytic evidence from randomized controlled trials confirms this pattern, with pooled mean differences of approximately 3.7–3.9 mmHg favoring SEVs from short-term to long-term follow-up [25].

The prompt relief of increased afterload with TAVI leads to an immediate improvement in cardiac performance, as evaluated via 2D echocardiography, which is subsequently followed by a reduction in LV volume and mass [26,27]. Consistent with previous studies, we found a notable improvement in LVEF on early post-TAVI echocardiography without a corresponding significant reduction in LV diameter. Bauer et al. attributed this immediate enhancement in LVEF following TAVI to the Frank–Starling mechanism. An immediate decrease in afterload leads to significant improvement in regional and global LV function as soon as TAVI, even in patients with a low ejection fraction [28]. Notably, this improvement results from the application of two-dimensional linear measurements for evaluation, and the use of three-dimensional volume measurements may show reverse remodeling.

Despite continuous advancements in transcatheter aortic valve design and implantation techniques, PVL remains a relatively frequent complication following TAVI. Contemporary data indicate that the incidence of mild AR was reported in about 29% of BEV and 36% of SEV recipients, whereas moderate-to-severe PVL was reported in approximately 0.8% of BEV and in 3.4% of SEV recipients [29,30]. In line with the literature, the frequency of PVL in our study tended to be higher in SEV patients compared to BEV patients and in SAV patients compared to IAV patients. PVL is associated with adverse clinical outcomes, particularly when severity progresses, likely reflecting subsequent left ventricular dilatation and dysfunction [31,32]. However, conflicting data have emerged regarding the prognostic implications of mild PVL. These discrepancies may stem from heterogeneity in baseline operative risk profiles across study populations, as well as inconsistencies in the grading methodologies employed for PVL assessment (ranging from three-grade to five-grade classification schemes) [32,33,34,35]. We used a four-class grading scheme. In our study, the observed incidence of AR aligned with previously published data.

The main limitation of this study is its retrospective and single-center design. The number of cases, especially in the BEVs, was relatively small, which limited the power to detect smaller differences between the groups. Invasive hemodynamic measurements are lacking, and correlation with echocardiographic findings would be an improvement. In addition, specific subsets, like bicuspid morphology affecting outcomes for valve hemodynamics, were missing. Another limitation is the lack of newer iterations of TAVI platforms (e.g., Evolut Pro, SAPIEN 3), which was due to institutional reimbursement restrictions. Annulus size data were not available for the present cohort, which precludes adjustment for this anatomical variable and limits the ability to definitively attribute the observed gradient differences between IAVs and SAVs to leaflet positioning per se, independent of annular geometry.

## 5. Conclusions

Modern TAVI valves provide significant relief in aortic valve obstruction. At the same time, variations among valves related to leaflet positioning and expansion mechanisms may affect early hemodynamic parameters. The current study has shown that TAVI with SAVs provides lower transaortic gradients compared to IAVs. On the other hand, the frequency of PVL was comparable between the valve groups. Future large-scale, randomized studies with extended follow-up are warranted to confirm these results and to clarify the long-term clinical implications of TAVI-valve-specific hemodynamic responses.

## Figures and Tables

**Table 1:** Detailed specifications of the transcatheter aortic bioprosthetic valve type used in our study.

Used Valves	No. of Patients Used	Valve Size	Expansion Mechanism	Leaflet Annular Position	External Skirt	Stent Frame	Leaflet
Myval	22 patients (6.6%)	23 mm—4 patients 24.5 mm—7 patients 26 mm—7 patients 27.5 mm—1 patient 29 mm—3 patients	Balloon expandable	Intra-annular	Yes	Nickel– cobalt	Bovine pericardium
Edwards SAPIEN XT Valve	35 patients (10.5%)	23 mm—9 patients 26 mm—24 patients 29 mm—2 patients	Balloon expandable	Intra-annular	No	Cobalt–chromium	Bovine pericardium
Portico	26 patients (7.8)	23 mm—1 patient 25 mm—6 patients 27 mm—11 patients 29 mm—8 patients	Self-expanding	Intra-annular	No	Nitinol	Bovine pericardium
CoreValve	54 patients (16.3%)	26 mm—16 patients 29 mm—28 patients 31 mm—10 patients	Self-expanding	Supra-annular	No	Nitinol	Porcine pericardium
Evolut R	195 patients (58.7%)	23 mm—4 patients 26 mm—59 patients 29 mm—99 patients 31 mm—1 patient 34 mm—32 patients	Self-expanding	Supra-annular	No	Nitinol	Porcine pericardium

**Table 2:** Baseline clinical and laboratory characteristics of the study population in terms of transcatheter aortic bioprosthetic valve type.

	Self-Expanding (*n* = 275)	Balloon-Expandable (*n* = 57)	*p*	Supra-Annular (*n* = 249)	Intra-Annular (*n* = 83)	*p*
Age (year)	78.3 ± 7.4	77.9 ± 6.6	0.711	78.3 ± 7.4	77.9 ± 7.0	0.630
Gender, female (%)	157 (57.1)	37 (64.9)	0.276	140 (56.2)	54 (65.1)	0.157
HT (%)	229 (83.3)	48 (84.2)	0.862	206 (82.7)	71 (85.5)	0.551
DM (%)	116 (42.2)	22 (38.6)	0.617	102 (41.0)	36 (43.4)	0.700
PAD (%)	23 (8.4)	2 (3.5)	0.276	23 (9.3)	2 (2.4)	0.040
COPD (%)	64 (23.4)	15 (26.3)	0.634	59 (23.8)	20 (24.1)	0.955
CABG (%)	58 (18.2)	11 (19.3)	0.843	46 (18.5)	15 (18.1)	0.935
Valve surgery (%)	13 (4.7)	2 (3.5)	1.000	13 (5.2)	2 (2.4)	0.373
PM implantation (%)	13 (4.7)	2 (3.5)	1.000	13 (5.2)	2 (2.4)	0.373
STS score (%)	4.2 (3.0–5.9)	3.8 (2.6–4.9)	0.056	4.2 (2.9–5.9)	3.9 (2.7–5.4)	0.360
Hemoglobin (g/dL)	11.2 ± 1.6	11.2 ± 1.6	0.994	11.2 ± 1.6	11.2 ± 1.7	0.862
WBC (×10^9^/L)	7.3 (6.1–8.8)	7.1 (5.6–8.8)	0.268	7.3 (6.1–8.7)	7.3 (5.7–8.8)	0.792
Platelet (×10^9^/L)	200.5 (165.0–262.3)	229.0 (164.5–255.0)	0.509	199.5 (165.8–260.8)	227.0 (164.0–256.0)	0.531
GFR (mL/min/1.73 m^2^)	66.0 (48.8–81.8)	57.0 (44.0–80.0)	0.219	66.0 (48.5–82.0)	59.0 (46.0–79.0)	0.256

HT, hypertension; DM, diabetes mellitus; PAD, peripheral artery disease; COPD, chronic obstructive pulmonary disease; CABG, coronary artery bypass graft; PM, pacemaker; STS, Society of Thoracic Surgeons; WBC, white blood cell; GFR, glomerular filtration rate.

**Table 3:** Baseline echocardiographic characteristics of the study population in terms of transcatheter aortic bioprosthetic valve type.

	Self-Expanding (*n* = 275)	Balloon-Expandable (*n* = 57)	*p*	Supra-Annular (*n* = 249)	Intra-Annular *(n* = 83)	*p*
LVEF (%)	52.4 ± 13.7	51.3 ± 12.8	0.578	52.3 ± 13.9	48.86 ± 12.52	0.886
LVEF ≤ 40% (%)	61 (22.3)	11 (19.3)	0.613	57 (23.1)	15 (18.1)	0.340
LVEDD (mm)	47.2 ± 7.2	47.8 ± 7.2	0.523	47.2 ± 7.3	47.6 ± 7.0	0.645
LVESD (mm)	32.2 ± 9.1	32.8 ± 9.7	0.669	32.4 ± 9.1	32.3 ± 9.5	0.989
IVS (mm)	14.4 ± 2.4	14.3 ± 2.2	0.833	14.3 ± 2.5	14.5 ± 2.2	0.468
PW (mm)	13.2 ± 2.0	12.8 ± 1.5	0.097	13.2 ± 2.1	12.9 ± 1.7	0.172
LA (mm)	44.2 ± 6.7	44.7 ± 5.7	0.624	44.3 ± 6.9	44.2 ± 5.5	0.914
PASP (mmHg)	42.0 ± 14.2	41.4 ± 12.6	0.749	41.6 ± 14.5	42.9 ± 12.0	0.456
Maximum AV gradient (mmHg)	74 (62–91)	70 (64–80)	0.136	74 (62–91)	71 (65–85)	0.451
Mean AV gradient (mmHg)	46 (39–57)	44 (40–51)	0.235	46 (39–57)	44 (40–53)	0.512
AVA (cm^2^)	0.6 ± 0.2	0.6 ± 0.1	0.517	0.6 ± 0.2	0.6 ± 0.1	0.492
MR	2.4 ± 0.7	2.40 ± 0.8	0.943	2.4 ± 0.7	2.5 ± 0.7	0.515
TR	2.5 ± 0.8	2.5 ± 0.7	0.975	2.5 ± 0.8	2.5 ± 0.7	0.987

LVEF, left ventricular ejection fraction; LVEDD, left ventricular end-diastolic diameter; LVESD, left ventricular end-systolic diameter; IVS, interventricular septum; PW, posterior wall; LA, left atrium; PASP, pulmonary systolic arterial pressure; AVA, aortic valve area; MR, mitral regurgitation; TR, tricuspid regurgitation.

**Table 4:** Postoperative echocardiographic variables according to the transcatheter aortic bioprosthetic valve type.

	Self-Expanding *(n* = 275)	Balloon-Expandable (*n* = 57)	*P*	Supra-Annular (*n* = 249)	Intra-Annular (*n* = 83)	*p*
LVEF (%)	54.7 ± 12.0	56.3 ± 9.9	0.352	54.6 ± 12.2	56.0 ± 10.1	0.311
LVEDD (mm)	46.9 ± 7.4	47.2 ± 6.7	0.810	47.0 ± 7.4	46.8 ± 6.8	0.850
LVESD (mm)	31.4 ± 8.8	31.1 ± 7.4	0.795	31.5 ± 8.9	30.8 ± 7.6	0.493
IVS (mm)	14.3 ± 2.4	14.3 ± 2.0	0.926	14.3 ± 2.4	14.5 ± 2.0	0.423
PW (mm)	13.2 ± 2.0	12.9 ± 1.4	0.390	13.2 ± 2.0	13.0 ± 1.6	0.583
LA (mm)	44.5 ± 6.6	44.8 ± 5.8	0.771	44.6 ± 6.8	44.4 ± 5.4	0.763
PASP (mmHg)	41.0 ± 14.4	40.9 ± 13.6	0.957	40.7 ± 14.6	42.0 ± 13.1	0.458
Maximum AV gradient (mmHg)	14 (10–20)	16 (13–21)	0.055	14 (10–20)	16 (13–21)	0.019
Mean AV gradient (mmHg)	8 (5–11)	8 (7–11)	0.121	8 (5–10)	9 (7–11)	0.014
AVA (cm^2^)	1.8 ± 0.4	1.8 ± 0.4	0.316	1.8 ± 0.3	1.8 ± 0.4	0.303
MR	2.4 ± 0.7	2.4 ± 0.6	0.945	2.3 ± 0.7	2.4 ± 0.6	0.411
TR	2.4 ± 0.8	2.5 ± 0.7	0.483	2.4 ± 0.8	2.5 ± 0.7	0.626

LVEF, left ventricular ejection fraction; LVEDD, left ventricular end-diastolic diameter; LVESD, left ventricular end-systolic diameter; IVS, interventricular septum; PW, posterior wall; LA, left atrium; PASP, pulmonary systolic arterial pressure; AVA, aortic valve area; MR, mitral regurgitation; TR, tricuspid regurgitation.

**Table 5:** The frequency of post-TAVI perivalvular leak grade according to the transcatheter aortic bioprosthetic valve type.

		Self-Expanding (*n* = 275)	Balloon-Expandable (*n* = 57)	*p*	Supra-Annular (*n* = 249)	Intra-Annular (*n* = 83)	*p*
Degree of PVL (%)	None/trivial	168 (61.1)	43 (75.4)		150 (60.2)	61 (73.5)	
	Mild	98 (35.6)	14 (24.6)	0.078	92 (36.9)	20 (24.1)	0.090
	Moderate	9 (3.3)	0 (0)		7 (2.8)	2 (2.4)	

PVL, perivalvular leak.

## Data Availability

The data presented in the study are available upon request from the corresponding author. The data are not publicly available due to the arrangements made by the Ethics Committee.

## Abbreviations

The following abbreviations are used in the manuscript:

AS	Aortic stenosis
TAVI	Transcatheter aortic valve implantation
MCV	Medtronic Core Valve
ESV	Edwards SAPIEN (or SAPIEN XT) valve
TTE	Transthoracic echocardiography
LFLG	Low-flow, low-gradient
EOA	Effective orifice area
LVEF	Left ventricular ejection fraction
STS	Society of Thoracic Surgeons Score
LVEDD	Left ventricular end-diastolic diameter
LVESD	Left ventricular end-systolic diameter
IVS	Interventricular septum
PW	Posterior wall
LA	Left atrium
PASP	Pulmonary artery systolic pressure
AVmax	Maximum aortic jet velocity
AVA	Aortic valve area
MR	Mitral regurgitation
AR	Aortic regurgitation
PVL	Perivalvular leak
TR	Tricuspid regurgitation
PHT	Pulmonary hypertension
